# Overuse of glucocorticoids in rheumatoid arthritis: a national survey of primary care physicians

**DOI:** 10.1093/rap/rky049

**Published:** 2018-11-23

**Authors:** Beth I Wallace, Akbar K Waljee, Arlene Weissman, Tanner J Caverly, Sameer D Saini

**Affiliations:** 1 Department of Internal Medicine, Division of Rheumatology, University of Michigan Health System; 2 Institute for Healthcare Policy and Innovation, University of Michigan Medical School; 3 Center for Clinical Management Research, VA Ann Arbor Health Care System; 4 Department of Internal Medicine, Division of Gastroenterology and Hepatology, University of Michigan Health System, Ann Arbor, MI; 5 American College of Physicians, Research Center, Philadelphia, PA; 6 Department of Internal Medicine, Division of General Medicine, University of Michigan Health System, Ann Arbor, MI, USA

**Key message**

- Primary care providers overprescribe glucocorticoids for RA management, perhaps owing to inadequate communication with rheumatologists.

Sir, RA generates >$39 billion annually in health and societal costs [1] and affects 1.3 million Americans. Most individuals with RA receive oral glucocorticoids, such as prednisone [2], despite growing concerns about their safety [3]. National guidelines recommend using glucocorticoids at the lowest possible dose and for the shortest possible duration to treat RA symptom flares [4]; however, no optimal exposure threshold for RA management has been defined because the risks of glucocorticoid exposure vary widely based on patient factors [5]. We sought to evaluate how primary care physicians (PCPs) prescribe glucocorticoids for RA flares in patients co-managed by rheumatologists. We hypothesized that a PCP’s perception of glucocorticoid side effects influences their willingness to use glucocorticoids for patients with established RA.

In 2017, we conducted an online, cross-sectional survey of American College of Physicians members, using its Internal Medicine Insider Research Panel. Eligible PCPs had no subspecialty training and spent ≥25% of their time in direct patient care. Institutional Review Board approval was obtained from the Institutional Review Boards of the University of Michigan Medical School (IRBMED), # HUM00101280. We developed a vignette featuring a hypothetical 62-year-old woman who experiences a severe RA flare. This hypothetical patient’s medical history was described as including hypertension, type II diabetes, hyperlipidaemia, chronic obstructive pulmonary disease and tobacco abuse. Her family history included osteoporosis in her mother, and early myocardial infarction in her father and brother. She was described as being followed by a rheumatologist who she had difficulty contacting during RA flares previously. Respondents selected management options for this hypothetical patient from a prepopulated list including laboratory tests, imaging studies and treatments including glucocorticoids, NSAIDs and DMARDs. Respondents willing to prescribe oral glucocorticoids to this hypothetical patient were asked how long they would continue treatment.

Respondents rated the degree to which patient factors affected willingness to prescribe glucocorticoids (Table 1) and the degree to which two different oral glucocorticoid regimens increased the risk of common glucocorticoid side effects (Table 2). We used multivariable linear regression to estimate the association between PCP perception of glucocorticoid side effects and the duration of glucocorticoids a PCP chose to prescribe.

**Table 1:** Effect of a patient’s glucocorticoid-sensitive co-morbidities on respondent willingness to prescribe glucocorticoids for RA management

	Less willing to prescribe [*n* (%)]	Would not change willingness to prescribe [*n* (%)]	More willing to prescribe [*n* (%)]
Patient has treated hypertension	35 (14)	206 (84)	3 (0)
Patient had a heart attack 1 year ago	40 (16)	202 (83)	2 (0)
Patient had a heart attack 10 years ago	8 (3)	233 (95)	3 (0)
Patient has a BMI of 40 kg/m^2^	63 (25)	177 (73)	4 (0)
Patient has treated hyperlipidaemia	11 (5)	230 (94)	3 (0)
Patient has an asymptomatic vertebral compression fracture seen on X-ray	145 (59)	98 (40)	1 (0)
Patient was recently hospitalized for hip fracture	169 (69)	74 (30)	1 (0)
Patient has osteopenia on bone densitometry scan	116 (48)	128 (52)	0 (0)
Patient’s mother fractured her hip	49 (20)	195 (80)	0 (0)
Patient has treated peptic ulcer disease	124 (51)	119 (49)	1 (0)
Patient received four courses of oral antibiotics for sinus infections last year	79 (32)	157 (64)	8 (3)
Patient was hospitalized for pneumonia 3 months ago	43 (17)	198 (81)	3 (1)
Patient has treated congestive heart failure	91 (37)	151 (62)	2 (0)

**Table 2:** Effect of patient demographic and social factors on respondent willingness to prescribe glucocorticoids for RA management

	**Less willing to prescribe [*n* (%)]**	**Would not change willingness to prescribe [*n* (%)]**	**More willing to prescribe [*n* (%)]**
Patient is on Medicaid or uninsured	0 (0)	113 (90)	13 (10)
Patient has commercial insurance or Medicare	0 (0)	123 (98)	3 (0)
Patient has difficulty attending scheduled follow-up appointments	46 (37)	62 (49)	18 (14)
Patient has difficulty affording necessities, such as food, clothing and housing	5 (4)	100 (79)	21 (17)
Patient has limited access to medical specialty services, such as pulmonology, rheumatology and haematology	2 (2)	74 (59)	50 (40)
Patient is a postmenopausal woman	9 (7)	114 (90)	3 (2)
Patient is a man >50 years of age	2 (2)	119 (94)	5 (4)
Patient is unemployed	1 (0)	113 (90)	12 (10)

Of 557 eligible physicians, 244 (44%) completed the survey. Their mean age was 51 (s.d. 10.6) years, 35% were female, 74% were in non-academic practice, and 51% practised in a suburban area. In the 6 months before the survey, 58% of respondents encountered a patient experiencing RA flare, and 61% prescribed at least one glucocorticoid course for RA management (mean 4.2 glucocorticoid courses among prescribers). Most (72%) respondents reported that patients in their panels waited ≤1 month from referral to see a rheumatologist.

Two hundred and five respondents (84%) prescribed glucocorticoids for the hypothetical patient in the vignette. Of these, 137 (67%) would prescribe a burst of >20 mg/day prednisone equivalent, followed by a taper. One hundred and twenty-four respondents (51%) would continue glucocorticoids for ≥4 weeks. Fifty-three respondents (22%) would continue for ≥6 weeks.

More than 50% of respondents were less willing to prescribe glucocorticoids to RA patients with peptic ulcer disease, osteoporosis or fractures, and >30% were less willing to prescribe to patients with congestive heart failure or recurrent minor infections. However, most respondents would not alter prescribing behaviour for a history of recent myocardial infarction, serious infection, hypertension or hyperlipidaemia (Table 1). Forty per cent of respondents were more willing to prescribe glucocorticoids for RA patients with poor access to specialty care, and 17% were more willing to prescribe to patients with problems affording basic necessities. Patient age, sex and menopausal status did not affect respondent prescribing (Table 2).

More than 70% of PCPs perceived glucocorticoid side effects (matrix presented in Table 3) as unlikely. In multivariable analysis adjusting for provider and practice characteristics, PCP perception of glucocorticoid side effects did not predict the duration of glucocorticoids a PCP would prescribe (*P* = 0.2).

**Table 3:** Respondent willingness to prescribe glucocorticoids for RA management: The below matrix was presented to survey participants, who rated the degree to which two oral glucocorticoid regimens (20mg prednisone daily for 2 weeks; 5mg prednisone daily for 4 months) increased risk of eleven side effects

	Not at all (0)	A little (1)	A lot (2)
Cataracts			
Fractures or osteoporosis			
Minor infections^a^			
Serious infections^b^			
Hypertension			
Death			
Congestive heart failure			
Hyperlipidaemia			
Stroke or heart attack			
Venous thromboembolism^c^			
Diabetes			

a For example, upper respiratory infection or complicated urinary tract infection.

b For example, sepsis or pneumonia requiring hospitalization.

c For example, deep vein thrombosis or pulmonary embolism.

In this national sample, 51% of PCPs would prescribe ≥1 month of glucocorticoids to an RA patient at high risk for glucocorticoid side effects, despite good access to rheumatology care among their patients. The PCP’s concern about glucocorticoid side effects did not predict the duration of glucocorticoids they would prescribe. PCPs would reduce glucocorticoid prescribing in response to certain patient risk factors (osteoporosis, congestive heart failure) but not to others (myocardial infarction, serious infection). The PCPs were more willing to prescribe glucocorticoids to patients with difficulty accessing specialty care or affording necessities.

Recent studies link even short-term glucocorticoid exposure to serious side effects, such as venous thromboembolism, fractures and sepsis. [3] Given these data, why are PCPs willing to prescribe prolonged courses of medications like glucocorticoids? In many cases, front-line providers may feel they have little choice, even when the patient is co-managed with a specialist. The PCPs and the specialists both believe that inadequate information from the other compromises their ability to provide patient care [6], and prior work has shown that direct, interactive communication between treating physicians improves patient outcomes [7]. When PCPs perceive treating specialists as inaccessible to themselves or their patients, they may feel obligated to treat uncontrolled chronic conditions themselves, perhaps relying on familiar, inexpensive medications, such as glucocorticoids, to avoid modifying costly specialty regimens with which they lack experience, such as DMARDs [8].

Our work suggests that PCPs are likely to overprescribe glucocorticoids for RA, even when patients have good access to specialist care and providers are aware of the risk profile of glucocorticoids. Regular, interactive communication between PCPs and rheumatologists might improve patient outcomes by reducing glucocorticoid overuse and related avoidable side effects.

*Funding*: This work was supported by the National Institutes of Health [4T32AR007080-38].

*Disclosure statement*: A.W. is the director of the American College of Physicians Research Center. The other authors have declared no conflicts of interest.

Survey data are available from the authors upon request.
